# Association between tenofovir plasma trough concentrations in the early stage of administration and discontinuation of up to five years tenofovir disoproxil fumarate due to renal function-related adverse events in Japanese HIV-1 infected patients

**DOI:** 10.1186/s40780-024-00343-z

**Published:** 2024-05-10

**Authors:** Hiroki Yagura, Dai Watanabe, Takao Nakauchi, Hiroyuki Kushida, Kazuyuki Hirota, Yasuharu Nishida, Munehiro Yoshino, Tomoko Uehira, Takuma Shirasaka

**Affiliations:** 1 Department of Advanced Medicine for HIV Infection, Institute for Clinical Research, NHO Osaka National Hospital, 2-1-14, Hoenzakaa, Chou-Ku, Osaka, 540-0006 Japan; 2 Department of Pharmacy, NHO Kyoto Medical Center, Kyoto, Japan; 3 Department of Pharmacy, NHO Osaka National Hospital, Osaka, Japan; 4 AIDS Medical Center, NHO Osaka National Hospital, Osaka, Japan

**Keywords:** Tenofovir, Plasma trough concentration, Renal dysfunction

## Abstract

**Background:**

The relationship between plasma tenofovir (TFV) concentration at the beginning of tenofovir disoproxil fumarate (TDF) administration and the development of renal dysfunction during long-term administration of TDF has not been demonstrated yet. The objective of the present study was to determine whether plasma TFV trough concentrations during early TDF administration could serve as an indicator of renal dysfunction when TDF is administered for long periods.

**Methods:**

We included 149 HIV-1 infected Japanese patients who were prescribed TDF. We investigated the relationship between plasma TFV trough concentrations and the rate of discontinuation due to the development of renal dysfunction for up to five years after the start of TDF administration. We also examined how the decrease in renal function over time due to TDF administration was related to factors associated with high TFV levels and plasma TFV trough concentrations.

**Results:**

The median TFV trough concentration in the TDF discontinuation group was 88 ng/mL, which was significantly higher (*p* = 0.0041), than that in the continuation group (72 ng/mL). Further, using an ROC curve, the cut-off value for TFV trough concentration at which TDF discontinuation was significantly high was found to be 98 ng/mL. Logistic multivariate analysis of factors associated with discontinuation of TDF due to renal function-related adverse events showed that being ≥ 50 years old (OR = 2.96; 95% CI, 1.01–8.64), having eGFR < 80 mL/min/1.73m^2^ at the start of TDF administration (OR = 5.51; 95% CI, 1.83–17.5), and TFV trough concentration ≥ 98 ng/mL (OR = 2.96; 95% CI, 1.16–7.60) were independent factors.

**Conclusions:**

The results suggested that the importance of measuring TFV concentrations to evaluate the risk of developing renal function-related adverse events during long-term TDF administration.

## Background

Since the nucleoside analog reverse-transcriptase inhibitor tenofovir (TFV) is poorly absorbed by the intestinal tract, improved formulations with better absorption through esterification are administered clinically [1, 2]. Recently, tenofovir disoproxil fumarate (TDF) has also been prescribed, but a new TFV prodrug called tenofovir alafenamide fumarate (TAF) is recommended in the guidelines. Moreover, TDF has been shown to be highly effective in preventing infections before and after exposure [3–5].

TFV is known to cause renal dysfunction through tubular cell mitochondrial toxicity [6]. It has been suggested that this decline in renal function is dependent on the duration of TDF administration [7]. Moreover, recovery of renal function has been reported to be difficult even after discontinuation of TDF in patients with a severe decline in renal function who have been taking the drug for a long period [7]. Furthermore, it has been reported that when patients treated with TDF developed acute kidney injury, the symptoms became more severe and the recovery of renal function was slower [8].

Reported risk factors for renal dysfunction due to TDF administration include low body weight, long-term TDF administration, old age, and concomitant use of lopinavir/ritonavir [9–11]. Previous studies have reported high plasma TFV concentrations during renal dysfunction [12]. However, how the TFV concentration at the start of TDF treatment relates to the effects of long-term TDF administration on renal function has not been demonstrated yet.

The objective of the present study was to determine whether plasma TFV trough concentrations during early TDF administration could serve as an indicator of renal dysfunction when TDF is administered for long periods.

## Methods

### Patients

In this study, we included patients who fulfilled all of the following conditions: 1) Patients who started antiretroviral therapy regimens containing TDF or other regimens containing TDF, from January 2007 to December 2011, at the NHO Osaka National Hospital; 2) Patients who were Japanese, infected with HIV-1, and at least 18 years old; 3) Patients with plasma TFV concentration measurements 20–28 h after administration from day 7 to day 180 after the start of TDF administration; 4) Patients who could be followed for five years after the start of TDF administration; 5) Patients whose interview confirmed that they had not forgotten to take their medication in the 7 days prior to the TFV concentration measurement. Patients who changed to regimens containing dolutegravir, cobicistat, and rilpivirine, which are drugs that may cause an apparent rise in Serum Creatinine (SCr) from drug transporter inhibition, during the observation period were excluded from the study.

### Measurement of trough plasma TFV concentration

Immediately after collection, the blood samples were centrifuged at 3,000 rpm for 10 min. The plasma was collected and stored at -30 °C until measurement. Blood TFV concentration was measured using high-performance liquid chromatography by following a previously reported method [13].

### Procedures

Patients who discontinued TDF due to renal function-related adverse events during the follow-up period were placed in the discontinuation group and those who continued TDF for five years were placed in the continuation group.

Data on basic patient demographics were collected from the medical records. Age, weight, and BMI on the day TDF was administered were measured. Comorbidities considered to be potential risk factors for decline in renal function were hypertension, diabetes mellitus, dyslipidemia, and hyperuricemia. These were considered as comorbidities when medications for these diseases were continuously prescribed before TDF administration was started. We obtained CD4 cell count and HIV-RNA copies (≤ 50 copies) when tenofovir plasma concentrations are measured. The estimated glomerular filtration rate(eGFR) was obtained at the start of TDF administration. The background and plasma TFV trough concentrations were compared between the discontinuation and continuation groups. In addition, annual changes in median eGFR levels in the continuation group were examined by dividing the TFV trough concentrations into quartiles. eGFR was calculated using SCr levels with the following formulas given by the Japanese Society of Nephrology [14]:

- Male: eGFR (mL/min/1.73 m^2^) = 194 × Scr^−1.094^ × Age.^−0.287^
- Female: eGFR (mL/min/1.73 m^2^) = 194 × Scr^−1.094^ × Age^−0.287^ × 0.739

The study protocol was reviewed and approved by the Institutional Review Board of the Osaka National Hospital (approval no. 13058). The procedures were carried out in accordance with relevant guidelines and regulations. All samples were collected after acquiring written consent.

### Statistical analysis

The relationship between patient demographic factors and discontinuation of TDF due to renal function-related adverse events and plasma TFV trough levels were examined using the Mann–Whitney U test, Chi-squared test and Fisher's exact test. The rates of TDF discontinuation due to renal function-related adverse events for each TFV trough concentration quartile were examined using the Cochran-Armitage test. The relationship between elevated TFV concentration and the discontinuation rate was examined, and the cut-off TFV trough concentration for TDF discontinuation was calculated using a receiver operating characteristic (ROC) curve. The optimal cut-off value from the ROC curve was determined based on the Youden index. Background factors associated with discontinuation of administration due to renal function-related adverse events were examined using univariate and logistic multivariate analyses. The relationship between plasma TFV trough concentrations and the decline in renal function over time after TDF administration was examined using the Steel–Dwass test. The significance level was set at 5%. Statistical analysis was performed using JMP software version 11.2.0 (SAS Institute Inc., Cary, North Carolina).

## Results

### Discontinuation of TDF due to renal dysfunction in Japanese HIV-1-infected patients

Table 1 shows the general background of the 149 included subjects, who were divided into those who discontinued TDF within 5 years (discontinuation group) and those who continued administration for at least 5 years (continuation group). The discontinuation group included 34 patients (23%), with a median duration until discontinuation of 967 days (interquartile range: 552–1491). While the median age of the patients in the discontinuation group was 45 years, that of the continuation group was 37 years, which was significantly younger (*p* < 0.001). In addition, there were fewer hepatitis B cases in the discontinuation group than that in the continuation group. There was no significant difference in the number of cases of trimethoprim/sulfamethoxazole given to prevent of pneumocystis pneumonia, which causes an apparent rise in SCr, and there were no cases in which a similarly acting combination drug was added during the observation period.

**Table 1:** Demographics of participants

	Discontinuation	Continuation	*p*-value
Participants (n, %)	34	115	
Age, years [median; IQR]	45 [39–56]	37 [33–44]	0.004
Males (n, %)	34 (100%)	114 (99%)	0.515
Body weight, kg [median; IQR]	58 [53–70]	62[54–69]	0.279
Body mass Index [median; IQR]	21.2 [19.4–23.3]	21.3[19.4–23.7]	0.896
Body surface area, m^2^ [median; IQR]	1.67 [1.56–1.78]	1.72 [1.61–1.82]	0.104
CD4 cell count, cells/μL [median; IQR]	268 [143–398]	272 [151–370]	0.599
Estimated glomerular filtration rate, mL/min/1.73m^2^ [median; IQR]	90[78–108]	100[87–114]	0.058
Participants with HIV-1-RNA level < 50 at time of sampling (n, %)	15(44%)	39(34%)	0.233
Hypertension	2(6%)	2(2%)	0.224
Diabetes mellitus	2(6%)	1(1%)	0.129
Dyslipidemia	1(3%)	1(1%)	0.406
Hyperuricemia	2(6%)	2(2%)	0.224
AIDS (n, %)	9(26%)	38(33%)	0.277
Use of Trimethoprim/sulfamethoxazole (n, %)	6(18%)	19(17%)	0.877
Duration until measurement of plasma concentration (Median, days)	28	28	0.614
HBV infection (n, %)	1(3%)	21(18%)	0.018
HCV infection (n, %)	1(3%)	6(5%)	0.497
Treatment naïve (n, %)	33(97%)	108(94%)	0.475
Use of antiretroviral agents (n, %)			
Atazanavir/ritonavir (n, %)	14(41%)	41(36%)	0.558
Lopinavir/ritonavir (n, %)	7(21%)	26(23%)	0.803
Darunavir/ritonavir (n, %)	8(23%)	19(17%)	0.351
Fosamprenavir/ritonavir( n, %)	2(6%)	15(13%)	0.203
Efavirenz (n, %)	0(0%)	3(2%)	0.457
Raltegravir (n, %)	3(9%)	11(9%)	0.599

*IQR* interquartile range, *AIDS* Acquired immune deficiency syndrome, *HBV* Hepatitis B Virus, *HCV* Hepatitis C Virus

### Relationship between TDF discontinuation due to renal function-related adverse events and plasma TFV trough concentrations

**Fig. 1:**
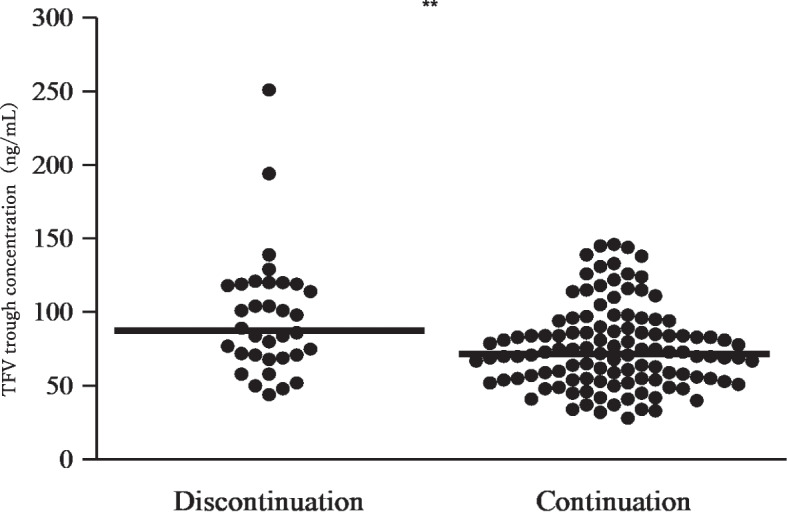
Comparison of Tenofovir plasma-trough concentrations and discontinuation due to impaired renal function. Horizontal straight line indicates median value. The Mann–Whitney U test showed significant differences (** *p* = 0.0041)

The median plasma TFV trough concentrations of all 149 patients was 75 ng/mL (interquartile range: 57–97). Figure 1 shows the TFV trough concentrations of the discontinuation and continuation groups. The median TFV trough concentration of the discontinuation group was 88 ng/mL, which was significantly higher (*p* = 0.0041) than that of the continuation group (72 ng/mL).

**Fig. 2:**
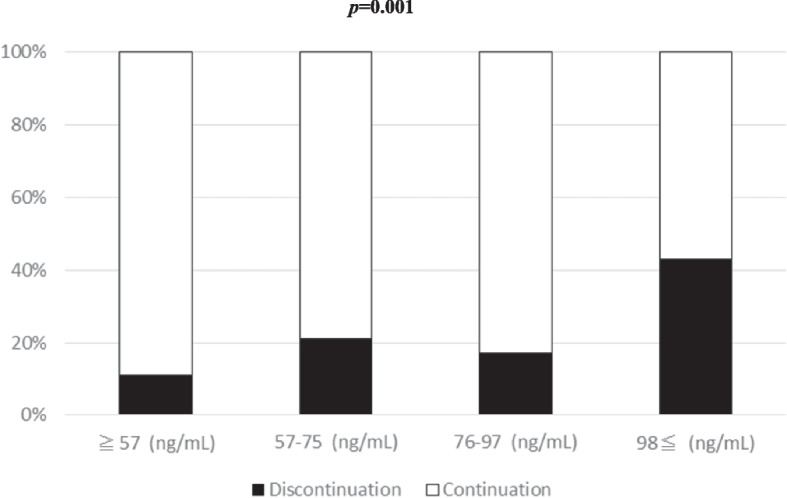
Association between trough Tenofovir concentration and discontinuation of TDF. *P* value by Cochran-Armitage test is shown

Figure 2 shows the rates of TDF discontinuation due to renal function-related adverse events for each TFV trough concentration quartile. The rate of TDF discontinuation tended to be higher when the TFV trough concentration was greater than or equal to the third quartile value (*p* = 0.001).

The cut-off TFV trough concentration for discontinuation of TDF calculated using an ROC curve was 98 ng/mL (AUC, 0.660; sensitivity, 0.471; specificity, 0.817).

### Factors associated with TDF discontinuation due to renal function-related adverse events

To investigate factors associated with the discontinuation of TDF due to renal function-related adverse events, univariate and logistic multivariate analyses were performed using explanatory variables, such as age ≥ 50 years, which is a previously reported risk factor for renal function decline due to TDF administration [10], eGFR levels < 80 (mL/min /1.73m^2^) at the start of TDF administration, median weight < 60 kg, and TFV trough concentration of ≥ 98 ng/mL, which is the cut-off value for TDF discontinuation calculated from the ROC curve. Hepatitis B was not listed as an explanatory variable because of the younger age (Median; 36 y.o.) and higher eGFR levels (Median; 105 mL/min /1.73m^2^) of the continuing patients. The Variance inflation factor (VIF) calculated by converting to dummy variables ranged from 1.1 to 1.2.

We found that being ≥ 50 years old (odds ratio (OR) 2.96; 95% confidence interval (CI), 1.01–8.64), having eGFR < 80 mL/min/1.73m^2^ (OR 5.51; 95% CI, 1.83–17.5), and having a TFV trough concentration of ≥ 98 ng/mL (OR 2.96; 95% CI, 1.16–7.60) were significant independent factors for TFV discontinuation due to renal function-related adverse events (Table 2).

**Table 2:** Associations between parameters and discontinuation of TDF

	Univariate results			Multivariate results		
	OR	95%CI	*p*-value	OR	95%CI	*p*-value
Age ≧ 50	5.73	2.20–14.9	0.0004	2.96	1.01–8.64	0.0481
Body Weight ≦ 60 kg	2.06	0.950–4.50	0.0784	2.13	0.825–5.81	0.1184
eGFR ≦ 80(mL/min/1.73m^3^)	5.15	2.02–13.2	0.0008	5.51	1.83–17.5	0.0025
TFV trough ≧ 98 ng/mL	4.48	1.97–10.2	0.0005	2.96	1.16–7.60	0.0236

*OR* odds ratio, *CI* confidence interval, *eGFR* estimated glomerular filtration rate, *TFV* tenofovir

### Relationship between plasma TFV trough concentration and decline in renal function over time due to TDF administration

**Fig. 3:**
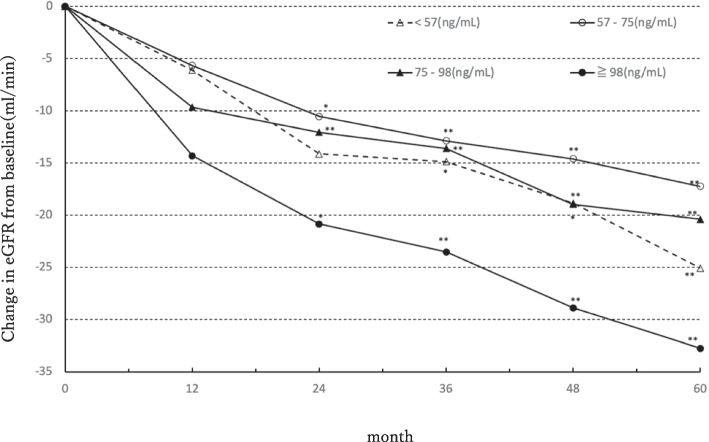
Association between trough Tenofovir concentration and median change in eGFR from baseline to 5 years. The x-axis is labeled with months to make the figure visually understandable; 30 days are used to represent 1 month. eGFR, estimated glomerular filtration rate. Filled circles, < 57 (ng/mL, *n* = 33); filled triangles, 57–75 (ng/mL, *n* = 28); filled diamonds, 75–98 (ng/mL, *n* = 33); filled squares, ≥ 98 (ng/mL, *n* = 21). The Steel–Dwass test showed significant differences (*p < 0.05, **p < 0.01)

The 115 patients in the continuation group were divided by the TFV trough concentration quartiles for all 149 cases to examine annual changes in eGFR from the start of TDF administration till after five years. Figure 3 shows the median decline in eGFR per year, from the start of TDF administration. In the TFV trough concentration quartile, after five years, eGFR declined by 25, 17, 20, and 31 mL/min, respectively. eGFR was significantly lower after three years for the first and second quartiles, and after one year for the third and fourth quartiles than that at the start of administration. Further, for the fourth quartile, eGFR level after five years was significantly lower than that after one and two years. Also, the fourth quartile coincidentally matched the cutoff value by ROC.

## Discussion

In the present study, TFV trough concentrations were significantly higher in patients who discontinued TDF due to renal dysfunction. Furthermore, among patients who continued TDF for five years, those with high TFV concentrations showed relatively early decline in renal function. This indicates that in addition to previous findings stating that renal function declines over time with continuous TDF administration [9], the TFV concentration is associated with the speed at which renal function declines and with the discontinuation of TDF due to renal function decline. This also suggests a possible association between plasma TFV trough concentrations and the amount of TFV exposure in renal tubular cells. We calculated a 98 ng/mL cut-off TFV trough concentration for the discontinuation of TDF due to renal dysfunction. However, this cannot simply be extrapolated to all cases, as drugs containing TDF are not only used for treatment purposes, but in recent years, these drugs are also used for pre-exposure prophylaxis (PrEP). The incidence of renal dysfunction with TDF administration in the case of PrEP has been reported to be lower than in HIV-infected patients [15]. Patients undergoing PrEP differ from the subjects of the present study in terms of the presence of HIV infection and the use of other anti-HIV drugs. Nevertheless, this cut-off value may be useful as a predictor of renal function decline.

Furthermore, even in cases with relatively low concentrations, although the rate of eGFR decline was slower than that of cases with high concentrations, in some cases, TDF administration was discontinued. TFV is taken up into tubular cells via organic anion transporters 1 and 3 present in the basolateral membrane of tubular cells [16], and is excreted by multidrug resistance-associated proteins 4 and 2 (MRP4, MRP2) on the apical site [17, 18]. TDF administration has been reported to be associated with reduced renal tubule function and genetic polymorphisms of the adenosine triphosphate-binding cassette, which encodes MRP2 [19]. This may help explain the presence of cases with low TFV concentrations and reduced eGFR. Therefore, long-term variations in renal function should also be examined with TAF, which results in plasma TFV concentrations lower than those with TDF and has been frequently used in recent years.

We also analyzed the association between TDF discontinuation and the combined use of protease inhibitors (PIs) boosted by ritonavir, but found no obvious link between the two (data not shown). Since it has been reported that switching from atazanavir or lopinavir to darunavir improves renal function even when PI is boosted by ritonavir [20], PIs that have a different effect on the kidneys may have been examined together.

The present study had several limitations. First, it was a retrospective, observational study with a limited number of subjects from a single center. The sample included only one female patient. In addition, we only examined trough concentrations of plasma TFV concentration, and renal function was assessed solely based on SCr and eGFR. Also, the possibility of multicollinearity cannot be ruled out for the results of the multivariate analysis, although the VIF showed a low value.

## Conclusion

In conclusion, this study suggests that the discontinuation of TDF due to renal function-related adverse events from long-term administration in Japanese HIV-1 infected patients is related to high blood TFV trough concentrations, in addition to advanced age and reduced renal function. This demonstrates the importance of measuring TFV concentrations to evaluate the risk of developing renal function-related adverse events during long-term TDF administration.

## Data Availability

The data used in this study were obtained under an exclusive data-sharing agreement and are not currently publicly available.

## Abbreviations

TFV: Tenofovir
TDF: Tenofovir disoproxil fumarate
TAF: Tenofovir alafenamide fumarate
SCr: Serum creatinine
eGFR: Estimated glomerular filtration rate
ROC: Receiver operating characteristic
OR: Odds ratio
CI: Confidence interval
VIF: Variance inflation factor
PrEP: Pre-exposure prophylaxis
MRP: Multidrug resistance-associated proteins
PIs: Protease inhibitors
